# Utilizing Longitudinal Measures of Fetal Growth to Create a Standard Method to Assess the Impacts of Maternal Disease and Environmental Exposure

**DOI:** 10.1371/journal.pone.0146532

**Published:** 2016-01-05

**Authors:** David E. Cantonwine, Kelly K. Ferguson, Bhramar Mukherjee, Yin-Hsiu Chen, Nicole A. Smith, Julian N. Robinson, Peter M. Doubilet, John D. Meeker, Thomas F. McElrath

**Affiliations:** 1 Department of Obstetrics and Gynecology, Brigham and Women’s Hospital, Boston, Massachusetts, United States of America; 2 Department of Environmental Health Sciences, University of Michigan School of Public Health, Ann Arbor, Michigan, United States of America; 3 Department of Biostatistics, University of Michigan School of Public Health, Ann Arbor, Michigan, United States of America; 4 Department of Radiology, Brigham and Women’s Hospital, Boston, Massachusetts, United States of America; The University of Tennessee Health Science Center, UNITED STATES

## Abstract

Impaired or suboptimal fetal growth is associated with an increased risk of perinatal morbidity and mortality. By utilizing readily available clinical data on the relative size of the fetus at multiple points in pregnancy, including delivery, future epidemiological research can improve our understanding of the impacts of maternal, fetal, and environmental factors on fetal growth at different windows during pregnancy. This study presents mean and standard deviation ultrasound measurements from a clinically representative US population that can be utilized for creating Z-scores to this end. Between 2006 and 2012, 18, 904 non-anomalous pregnancies that received prenatal care, first and second trimester ultrasound evaluations, and ultimately delivered singleton newborns at Brigham and Women’s hospital in Boston were used to create the standard population. To illustrate the utility of this standard, we created Z-scores for ultrasound and delivery measurements for a cohort study population and examined associations with factors known to be associated with fetal growth. In addition to cross-sectional regression models, we created linear mixed models and generalized additive mixed models to illustrate how these scores can be utilized longitudinally and for the identification of windows of susceptibility. After adjustment for *a priori* confounders, maternal BMI was positively associated with increased fetal size beginning in the second trimester in cross-sectional models. Female infants and maternal smoking were associated with consistently reduced fetal size in the longitudinal models. Maternal age had a non-significant association with increased size in the first trimester that was attenuated as gestation progressed. As the growth measurements examined here are widely available in contemporary obstetrical practice, these data may be abstracted from medical records by investigators and standardized with the population means presented here. This will enable easy extension of clinical data to epidemiologic studies investigating novel maternal, fetal, and environmental factors that may impact fetal growth.

## Introduction

Impaired or suboptimal fetal growth is associated with an increased risk of perinatal morbidity and mortality as well as an increased risk of later life conditions including type II diabetes, hypertension, obesity and impaired neurodevelopment [1–5]. The epidemiologic evaluation of predictors of fetal growth has typically involved the assessment of birth weight alone, birth weight for gestational age at delivery, and in some cases the use of other anthropometric measures, such as infant head circumference at birth. However, these studies do not adequately consider the time points during pregnancy when growth is slowed or affected. Identifying these specific windows has the potential to improve the understanding of how both endogenous and exogenous factors potentially influence fetal development, which could in turn inform intervention and prevention strategies.

A second major issue with studies of fetal growth is that they are frequently underpowered, though we acknowledge smaller studies may be unavoidable if the study aims require examination of expensive biomarkers or similar cost prohibitive tests. These limitations, however, are not insurmountable.

In the present paper, we present mean and standard deviations of ultrasound measurements and birth weight from an unbiased and clinically representative population of pregnant women who delivered at Brigham and Women’s Hospital (BWH) in Boston, Massachusetts, USA, from 2006–2012. Measures from this standard population can be utilized in epidemiologic studies of longitudinal fetal growth, and may be particularly useful for researchers with populations in the US. We also illustrate the epidemiologic utility of these data by creating Z-scores for measures from a cohort study population (N = 868) and examining the effects of maternal age, maternal BMI, fetal gender, and maternal tobacco use during pregnancy on fetal growth across gestation.

## Materials and Methods

### The standard population

The American College of Obstetricians and Gynecologists’ (AJOG) current standards for prenatal care suggest that all women undergo ultrasound evaluations both at the end of the first trimester, for the estimation of aneuploidy risk, and then again at the mid-point of the second trimester to assess fetal anatomy [6]. Information abstracted from these measurements, therefore, represents an unbiased source of data since nearly all women are required to undergo these exams.

Between 2006 and 2012 there were 18, 904 non-anomalous pregnancies which received prenatal care, first and second trimester ultrasound evaluations, and ultimately delivered singleton newborns at BWH. The only requirements for inclusion in this analysis were: 1) a live singleton birth; and 2) that the delivery occurred at BWH. (Note: this differs from the cohort study population, which required that women receive their prenatal care at the BWH academic practice.) Crown rump length (CRL) was abstracted from the first trimester aneuploidy screening ultrasound. Subsequently, occipito-frontal diameter, head circumference, abdominal circumference, biparietal diameter, and femur length were abstracted from the second trimester morphology survey. Infant weight at delivery was abstracted from the electronic delivery record. All ultrasound measurements were made by faculty of the Radiology and Maternal-Fetal Medicine Departments at BWH, who are all experienced sonologists with active Society of Maternal-Fetal Medicine certification. Patients with more than one qualifying pregnancy between 2006 and 2012 contributed data from a gestation chosen at random. Dating was established in a two-step hierarchical fashion consistent with current AJOG recommendations [6]. First, in cases of *in vitro* fertilization (IVF) the estimated date of confinement (EDC) was fixed based on the known date of conception. Second, last menstrual period (LMP) was used if it agreed with the first trimester ultrasound (i.e., if dating by LMP differed by no more than 8% from that determined by ultrasound). If the LMP was unknown or was inconsistent with the first trimester ultrasound, the first trimester ultrasound estimate was used for gestational dating.

The crown rump lengths from the standard population were stratified by the week of measurement for weeks 9 through 12. Fetal anthropometric measurements from the second trimester were stratified for weeks 16–22. These measurements included: abdominal circumference (mm), biparietal diameter (mm), femur length (mm), occipitofrontal diameter (mm), and head circumference (mm). In addition to individual measurements, we also calculated estimated fetal size (g) at the second trimester using the formula of Hadlock, which combines biparietal diameter, abdominal circumference, and femur length [7]. Birth weight (g) was stratified by week of gestation at delivery (weeks 23 to 42). Means and standard deviations were calculated for each metric for the relevant weeks of gestation (Table 1).

**Table 1:** Mean and Standard Deviations for Brigham & Women’s Hospital Standard Population.

**Observation Time 1**														
	Crown rump length (mm)													
Gestational age (weeks)	Mean		SD											
9	26.32		3.75											
10	36.37		4.40											
11	47.52		4.88											
12	58.88		4.81											
13	68.73		3.92											
**Observation Time 2**														
	Abdominal diameter (mm)		Abdominal circumference (mm)		Biparietal diameter (mm)		Femur length (mm)		Head circumference (mm)		Occipitofrontal diameter (mm)		Estimated fetal weight (g)	
Gestational age (weeks)	Mean	SD	Mean	SD	Mean	SD	Mean	SD	Mean	SD	Mean	SD	Mean	SD
16	34.21	2.55	107.44	8.12	34.85	1.95	21.05	1.97	126.12	6.78	43.43	2.47	181.62	20.88
17	37.98	2.66	119.27	8.55	38.26	2.02	24.43	1.97	139.22	7.02	47.93	2.56	222.84	25.27
18	41.58	2.81	130.58	9.04	41.34	2.10	27.58	1.98	151.43	7.35	52.25	2.67	269.33	30.63
19	45.25	3.04	142.08	9.89	44.32	2.23	30.59	2.08	163.48	7.66	56.53	2.83	324.14	38.30
20	49.04	3.28	154.02	10.55	47.37	2.42	33.61	2.26	175.77	8.28	60.93	3.02	389.74	48.01
21	52.81	3.50	165.82	11.40	50.47	2.59	36.59	2.46	188.10	8.87	65.22	3.24	464.75	59.55
22	56.54	3.71	177.53	12.12	53.58	2.72	39.44	2.53	200.13	9.36	69.44	3.38	550.04	72.07
**Observation Time 3**														
	Birth weight (g)													
Gestational age (weeks)	Mean		SD											
23	552.64		85.26											
24	641.22		103.21											
25	729.78		133.61											
26	828.78		165.57											
27	958.75		204.03											
28	1113.04		250.89											
29	1257.19		281.05											
30	1440.98		310.05											
31	1614.55		338.42											
32	1833.60		380.91											
33	2052.38		395.30											
34	2312.06		411.60											
35	2571.04		422.52											
36	2822.02		442.67											
37	3062.61		447.12											
38	3272.88		440.36											
39	3443.03		434.53											
40	3564.76		426.60											
41	3653.07		415.57											
42	3667.90		409.54											

### The cohort study population

The study population was derived from a pregnancy cohort, begun in 2006, that prospectively enrolls women seeking care at the faculty, midwifery, or resident practices of BWH. Women are enrolled early in the first trimester (median 10 weeks gestation) and followed until delivery. Women are eligible for participation in the study if they are at least 18 years of age, seek prenatal care prior to 15 weeks gestation, and plan to deliver at the BWH hospital. Thus, this sample is a subset of the standard population; however we ensured that none of the cohort study participants were included in the standard population.

At enrollment, each woman completes a survey to provide demographic, socioeconomic, and behavioral information. At delivery, medical history and events during the pregnancy were abstracted from medical records. CRL, second trimester ultrasound measurements, and birth weight were also abstracted from the clinical record for each patient in the cohort. Estimated due date was established using the same hierarchy as described above. Body mass index (BMI; kg/m^2^) was calculated based on height and weight at the first prenatal visit. Maternal age was also taken from the time of the first prenatal visit. Maternal smoking status was positive (dichotomized as Yes/No) if the patient reported any cigarette use that persisted throughout pregnancy. Only singleton non-anomalous pregnancies were included in this analysis. The study protocol was approved by institutional review board at BWH, and written informed consent was obtained from all participating women.

### Z-score calculations

Using means and standard deviations from the Standard Population stratified by gestational week, Z-scores were calculated for CRL (Observation Time 1), estimated fetal size (Observation Time 2) and birth weight (Observation Time 3) for each subject in the Cohort Study Population. The calculation was made in the following fashion:

$$Z=\frac{Observed\left(i\right)-sMean\left(i\right)}{sStandard\;Devation\left(i\right)}$$

where *Observed* refers to the metric under consideration at week *i* for the study subject, *sMean* refers to the Standard Population mean for that metric at week *i*, and *sStandard Deviation* refers to the standard deviation of the metric at week *i* from the Standard Population. We were thus able to create a longitudinal series of size Z-scores for our Cohort Study Population corresponding to the Observation Times 1 to 3.

### Statistical analysis

First, we performed cross-sectional analysis to examine the relationship between Z-scored fetal growth measurements at each Observation Time 1 (1^st^ trimester ultrasound), 2 (2^nd^ trimester ultrasound), and 3 (delivery) in association with maternal factors of interest, including maternal age, BMI, fetal gender, and maternal smoking status. Linear regression models were additionally adjusted for maternal race/ethnicity, health insurance provider, alcohol use during pregnancy, use of IVF for conception, gestational age at growth measurement, and diagnosis of preeclampsia in current pregnancy *a priori*.

Second, we performed a longitudinal analysis, examining fetal growth Z-scores from each observation time in the same model. We combined Z-scores of CRL (Observation Time 1), estimated fetal weight (Observation Time 2), and birth weight (Observation Time 3) into one vector for this analysis. Using a generalized least squares model with unstructured residual correlation, we regressed the Z-score vector on gestational age at ultrasound and the variables examined in the cross-sectional analysis. Z-score vector and gestational age at ultrasound were treated as time-varying factors and covariates were examined as fixed effects. The model implemented was as follows:

$$y_{ij}=\beta_{0}+\beta_{1}x_{1ij}+\beta_{2}x_{2i}+\beta_{3}x_{3i}+\beta_{4}x_{4i}+\beta_{5}x_{5i}+\beta_{6}^{T}\;z_{i}+\varepsilon_{ij}$$

$$\varepsilon_{i}={\left(\varepsilon_{i1},\varepsilon_{i2},\varepsilon_{i3}\right)}^{T}∼MVN\left(0,\sigma^{2}\Sigma \left(\alpha \right)\right)$$

$$\Sigma \left(\alpha \right)=\left[\begin{array}{c} \;1\;\alpha_{12}\;\alpha_{13}\\ \alpha_{12}\;1\;\alpha_{23}\\ \alpha_{13}\;\alpha_{23}\;1\; \end{array}\right]$$

where *y*_*ij*_ is Z-scored fetal growth measurement for subject *i* at time *j*, *x*_1*ij*_ is gestational age (centered at 20 weeks) for subject *i* at time *j*, *x*_2*i*_,*x*_3*i*_,*x*_4*i*_, and *x*_5*i*_ are maternal age, BMI, fetal gender, and maternal smoking status for subject *i*, respectively, and ***z***_***i***_ is the vector of the additional covariates for subject *i*. The error term ***ε***_***i***_ is defined as shown with a multivariate normal distribution and an unstructured correlation matrix for the within-subject correlation of the repeated Z-score measures on the same subject (***Σ***(***α***)). In subsequent models we examined interaction terms between either fetal gender or maternal smoking during pregnancy and gestational age at the time of measurement. Gestational age was centered at 20 weeks for interpretability of interaction terms.

Third, to explore potential non-linear association between measures of fetal growth and other covariates of interest (including gestational time) we used a generalized additive mixed model (GAMM) with an unstructured correlation structure. The intent of this analysis is to examine the relationship between gestational age and fetal growth in a non-linear fashion with potential effect modifiers maternal age, maternal BMI, fetal gender, or maternal smoking status, so that associations can be examined visually and sensitive time points when fetal growth measurements are most strongly impacted can be identified. Three possible interaction structures between gestational age and these covariates (nonlinear interaction, linear interaction, and no interaction) were examined and the best-fit structure based on Bayesian information criterion was incorporated into the final GAMM. The model with nonlinear interaction terms was defined as:

$$y_{ij}=\beta_{0}+g_{1}(x_{1ij})+g_{2}(x_{1ij})x_{2i}+g_{3}(x_{1ij})x_{3i}+g_{4}(x_{1ij})x_{4i}+g_{5}(x_{1ij})x_{5i}+\beta_{6}^{T}\;z_{i}+\varepsilon_{ij}$$

where *g*_1_(.),*g*_2_(.),*g*_3_(.),*g*_4_(.), and *g*_5_(.) are smooth functions for the relationship between the gestational age and each of the non time-varying covariates of interest. The random effects were defined the same as in the marginal model with an unstructured correlation matrix.

All statistical analyses were performed using R version 3.0.2 (R Foundation for Statistical Computing, Vienna, Austria) and SAS version 9.2 (SAS Institute Inc., Cary, NC). P-values less than 0.05 were deemed statistically significant.

## Results

### The standard population

Characteristics of the Standard Population (N = 18,904) are representative of singleton patients seeking care at BWH over this interval. Mean maternal age was 32 years. Primiparous births represented 47% of the population. Over half of the population (55%) self-reported as White, while approximately 17%, 15%, and 10% self-reported as African-American, Hispanic, or Asian, respectively. Means and standard deviations from the Standard Population stratified by gestational week are presented in Table 1, where Observation Time 1 (weeks 9–13) presents CRL, Observation Time 2 (weeks 14–22) presents individual and summed (i.e., estimated fetal weight) anthropometric measurements, and Observation Time 3 (weeks 23–42) presents birth weight only. We additionally calculated means and standard deviation of anthropometric measurements from ultrasound scans taken between weeks 23–42. Because these scans were taken outside the clinically proscribed window for second trimester assessment of fetal anatomy, these values are presented as a supplement (S1 Table). While these measurements are more likely to be from pregnancies with suspected complications, they still may be of utility as a reference.

### The cohort study population

Characteristics of the Cohort Study Population (N = 868) are presented in Table 2. The Cohort Study Population was largely white (61.8%), had attained greater than a high school level education (85.1%), and were similar in age to the Standard Population (mean age 32.1 years). Among the Cohort Study Population subjects, 26 (3.0%) indicated they smoked during pregnancy and 435 (50.1%) delivered male infants. All of participants included in the Cohort Study Population had growth measurements available for each of the three Observation Time points.

**Table 2:** Summary Statistics of Cohort Study participants (N = 868). Means (standard deviations) are reported for continuous variables and counts and frequencies are reported for categorical variables.

Characteristic	N	Mean (SD) and [Range] or N (%)
Age (years)	859	32.1 (5.6) [18.3–50.2]
BMI at initial visit (kg/m^2^)	856	26.0 (5.9) [17.0–54.2]
Race/ethnicity	868	
White		536 (61.8%)
African-American		118 (13.6%)
Hispanic		126 (14.5%)
Other		88 (10.1%)
Insurance Status^a^	844	
Private/HMO		671 (79.5%)
Medicaid/SSI/Mass Health		173 (20.5%)
Education Level	855	
less than high school		33 (3.9%)
high school or GED equivalent		94 (11.0%)
greater than high school		728 (85.1%)
Nulliparous	868	363 (41.8%)
Smoked during pregnancy	868	26 (3.0%)
Drank alcohol during pregnancy	853	35 (4.1%)
Diabetes	868	24 (2.8%)
Gestational diabetes	868	68 (7.8%)
Chronic hypertension	868	58 (6.7%)
Preeclampsia	868	76 (8.8%)
Gestational age at delivery	868	38.6 (2.3) [28.4–42.7]
Infant Sex (% Male)	868	435 (50.1%)

^a^Abbreviations: Health maintenance organization (HMO), supplemental security income (SSI), general educational development (GED).

### Cross-sectional analysis

Table 3 demonstrates the cross-sectional associations between pregnancy characteristics of interest (maternal age, BMI, smoking status, and infant gender) Z-scores at each of the observation times. Models were adjusted *a priori* for variables known to influence fetal size, including maternal race/ethnicity, health insurance provider, alcohol use during pregnancy, use of IVF for conception, gestational age at observation time, and diagnosis of preeclampsia in current pregnancy. At the first trimester measurement (Observation Time 1), no significant associations were observed between the characteristics of interest and raw or Z-scored growth measurements, although maternal age was suggestively associated (p = 0.09) with the Z-scored measure (one year increase in maternal age associated with 0.018 mm increase in crown rump length). At the second trimester measurement (Observation Time 2) maternal BMI was positively associated with both raw (one kg/m^2^ increase associated with 1.56 gram increase in estimated fetal size) and Z-score (one kg/m^2^ increase associated with 0.017 increase in estimated fetal weight) measures, and female infants had significantly lower raw and Z-score growth indicators as well. At delivery (Observation Time 3), infant gender and smoking were inversely associated with raw as well as Z-score growth measures, and maternal pre-pregnancy BMI was positively associated with the Z-score growth measure.

**Table 3:** Cross-sectional associations between pregnancy characteristics and fetal growth measures at Observation Times 1–3. Growth measurements are modeled both as A) Raw growth measurements, and B) Z-scored growth measurements to the Standard Population.

Table 3A.									
	Observation time 1			Observation time 2			Observation time 3		
Variables of interest	β	SE	p	β	SE	p	β	SE	p
Maternal age	-0.133	0.129	0.304	0.637	0.373	0.088	-0.474	4.565	0.917
Maternal BMI	-0.156	0.110	0.155	1.564	0.318	0.000	6.756	3.901	0.084
Infant gender	-1.336	1.210	0.270	-7.500	3.495	0.032	-87.482	42.459	0.040
Maternal smoking	-5.265	3.634	0.148	8.674	10.602	0.414	-369.110	129.178	0.004
Table 3B.									
	Observation time 1			Observation time 2			Observation time 3		
Variables of interest	β	SE	p	β	SE	p	β	SE	p
Maternal age	0.018	0.010	0.090	0.003	0.007	0.704	-0.002	0.007	0.758
Maternal BMI	-0.002	0.009	0.845	0.017	0.006	0.003	0.026	0.006	0.000
Infant gender	0.040	0.098	0.682	-0.247	0.065	0.000	-0.227	0.070	0.001
Maternal smoking	0.098	0.294	0.739	0.312	0.197	0.114	-0.543	0.212	0.010

Note: Observation time 1 corresponds to crown rump length; Observation time 2 corresponds to estimated fetal weight; Observation time 3 corresponds to birth weight. Maternal age and BMI modeled continuously. Reference categories for categorical variables are as follows: Infant gender (male); Maternal smoking during pregnancy (none). Additional covariates for statistical models include: race/ethnicity, insurance status, alcohol use, use of IVF for conception, gestational age, and diagnosis of preeclampsia in current pregnancy.

### Longitudinal analysis: linear interaction

Results from the linear longitudinal analysis using the stacked Z-score fetal growth measures are presented in Table 4. Linear mixed models were adjusted for the same set of covariates as were included in linear regression models. Maternal age or maternal BMI did not show any interaction with gestational age. Model 1 included an interaction between infant gender and gestational age at Observation Time and Model 2 included an interaction between maternal smoking and gestational age at Observation Time. A suggestive (p = 0.11) interaction was observed between infant gender and gestational age in Model 1, indicating that female infants had slightly lower Z-score fetal growth profiles across the entire gestation compared to males. In Model 2, the interaction between maternal smoking and gestational age was statistically significant (p = 0.002), indicating that fetuses of mothers who smoked during pregnancy had significantly lower Z-score growth profiles across all points of gestation compared to mothers who did not smoke. Notably, these results indicate significant linear differences in growth across pregnancy in these groups.

**Table 4:** Adjusted associations between pregnancy characteristics and repeated measures of fetal growth (stacked Z-scores from observation times 1–3). Estimates derived from linear mixed effects models to adjust for within-subject correlation of measures.

	Model 1		
Variables of interest	β	SE	p
Maternal age	0.004	0.005	0.448
Maternal BMI	0.018	0.004	< .0001
Infant gender	-0.174	0.050	0.001
Maternal smoking	-0.050	0.142	0.727
Infant gender*gestational age	-0.005	0.003	0.112
	Model 2		
Variables of interest	β	SE	p
Maternal age	0.004	0.005	0.453
Maternal BMI	0.018	0.004	< .0001
Infant gender	-0.200	0.047	< .0001
Maternal smoking	0.089	0.149	0.552
Maternal smoking*gestational age	-0.032	0.010	0.002

Note: Both models included maternal age, maternal BMI, infant gender, and maternal smoking as variables of interest, as well as the following covariates: race/ethnicity, insurance status, alcohol use, use of IVF for conception, gestational age, and diagnosis of preeclampsia in current pregnancy. Additionally, each model contained one interaction term between a variable of interest and gestational age (centered at 20 weeks) of fetal growth measurement.

### Longitudinal analysis: non-linear interaction

**Fig 1:**
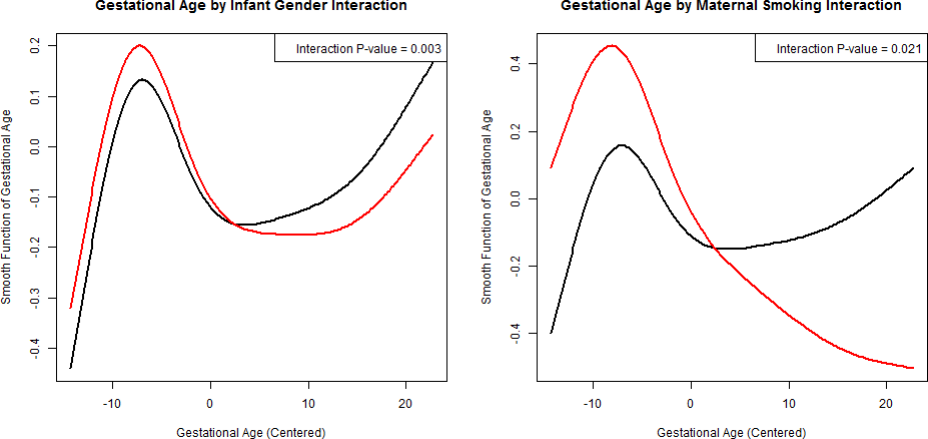
Generalized additive models (GAM) with an unstructured correlation structure between gestational age and fetal growth interacted by fetal gender (Red = Female, Black = Male) and maternal smoking status (Red = Smoking, Black = No Smoking).

To examine potential non-linear differences in growth trajectories by maternal smoking and fetal gender, we created generalized additive models. Again, covariates were consistent with those included in linear regression and linear mixed models. Model 1 included an interaction term between infant gender and gestational age at Observation Time, and Model 2 included an interaction term between maternal smoking and gestational age at Observation Time. Predicted values for fetal growth Z-score in association with gestational age by fetal gender and maternal smoking status are presented in Fig 1. For fetal gender and maternal smoking status, differences in fetal growth Z-scores across groups appeared to be non-linear. Male and female fetuses were similar early in pregnancy but as gestation progressed decreased growth in females became apparent (p for interaction = 0.003). Fetuses of smokers had slightly higher fetal growth Z-scores early in gestation, but toward the end of gestation fetal growth scores dropped to far below those from non-smoking mothers. Smaller p-values for GAM compared to linear mixed model interaction terms indicate the improved ability for the non-linear model to detect significant differences in growth Z-score trajectories between groups.

## Discussion

In this study we present means and standard deviations of first and second trimester ultrasound estimates of fetal size and corresponding birth weights in pregnancies ending in singleton live births from a large contemporary North American clinical service. We suggest that these can be utilized by researchers who have an interest in leveraging data that are automatically available in the clinical setting, because of their routine inclusion in prenatal care. These 1^st^ and 2^nd^ trimester ultrasound as well as birth weight measurements can be abstracted from medical records and standardized to Z-scores using the means and standard deviations presented here. Thus, these clinical data can be utilized for investigation of research questions pertaining to maternal, fetal, or environmental factors that may influence fetal growth. Additionally, as these measures are collected at multiple time points, they may be used for identification of windows of susceptibility during pregnancy when these factors may be impacting growth. We illustrate how these data may be utilized by presenting associations with maternal age, BMI, and smoking status, as well as fetal gender, in a Boston birth cohort with growth measurements standardized to this population.

Several other groups have performed large studies to collect ultrasound and birthweight data longitudinally for the creation of reference populations for standardization purposes. One example is from the INTERGROWTH-21^st^ project, designed specifically to create international reference curves for fetuses [8]. The standards were created from ultrasound scans and birth measurements taken from 4,321 pregnancies in 8 countries, and had very stringent exclusion criteria so that the population would represent healthy pregnancies with very low risk of adverse outcomes. Another example is the Generation R study (Rotterdam, the Netherlands) [9], a prospective birth cohort of 8,800 pregnancies. Our reference population differs from these notably because it represents a clinically representative population of patients receiving routine care, rather than subjects recruited and willing to participate in a research cohort. On a similar note, scans from our reference population are not selected to represent only healthy pregnancies (as in the INTERGROWTH-21^st^ project) and are thus more representative of realistic fetal growth. This difference means that Z-scores calculated from our standard will be less precise, and calculations of differences will have lower power. However, using this more heterogeneous population will provide better control for Type I error; i.e., differences in Z-scores detected will be less likely to be spurious. Another difference and advantage of our population is that it is specifically representative of growth in the US which may differ slightly from European populations. To our knowledge, this study is the largest to present ultrasound scans longitudinally across pregnancy to date in the US.

Recalling that Observation Time 1 (the ultrasound evaluation for CRL) corresponds to the end of the first trimester, Time 2 to the latter portion of the second trimester when the fetal survey is performed and Time 3 to delivery, our analysis indicates a variety of growth responses to the several exposures and conditions we have examined. In cross-sectional analysis of an inherently maternal condition (BMI), increased maternal BMI was significantly associated with increased estimated fetal size (Time 2) during the second trimester and birth weight, but not CRL (Time 1) in the first trimester. Longitudinal modeling did not support any alteration of this effect uniformly over time. Similar associations have been found by past researchers for birth weight and fetal growth parameters later in gestation [10–14].

When assessing an inherent fetal condition (fetal gender) we found differences in measurements taken *in utero* as well as in the gender specific pattern in birth weight that has been extensively supported in past literature [15–19]. This difference appeared to develop between 16 to 22 weeks of gestation but was absent in the initial CRL measurement. Longitudinal modeling suggests that this association may also be enhanced with increased gestational age, since this pattern follows a non-linear relationship.

An exogenous source of exposure, maternal cigarette smoking, demonstrates a different pattern with regard to fetal growth. In our cross-sectional analysis the findings that decreases in birth weight, but not estimated fetal size or CRL, are related to maternal cigarette smoking during pregnancy parallel findings in multiple prior studies [20–23]. We also show, in longitudinal models, a consistent negative interaction term with gestational age and cigarette smoking. This finding suggests that with increased gestational age, an increasingly stronger adverse association with maternal smoking is observed. Past studies that have assessed ultrasound-based growth parameters at varying time points during pregnancy find significant adverse effects with cigarette smoking clustered more consistently later in pregnancy as opposed to earlier [24–25]. It has been hypothesized that the reduction in birth weight or late pregnancy fetal growth measured associated with smoking may be related to developmental adaptations in placental vasculature and/or fetal arterial resistance [25–26].

Knowing the timing of an effect on fetal growth offers insights into possible underlying mechanisms. Lin & Santolaya-Forgas divide fetal cell growth into three phases [27]. The initial phase broadly corresponds with the embryonic period and extends to 16 weeks. This phase is characterized by cellular hyperplasia and involves a rapid increase in overall cell number. The second phase ranges from 16 to 32 weeks and consists of continued cellular hyperplasia but now with increasing hypertrophy of cellular size. The final phase occurs after 32 weeks when cellular numbers are broadly set and hypertrophy of cellular size increases. It is during this final phase that an increase in cellular glycogen and fetal fat deposition occurs. The rate of fetal growth increases with each of these phases from 5g per day at 15 weeks, 20g per day at 24 weeks to 35g per day at 34 weeks [28]. Different fetal and maternal characteristics or exogenous exposures are unlikely to affect these growth phases equivalently. Examination between such characteristics or exposures and fetal Z-scores will indicate the portion of pregnancy where the effect manifests and thus indicate a possible mechanism. The adverse effects of exposure to cigarette smoke manifest in later pregnancy, suggesting that cellular hypertrophy and possibly glycogen deposition is preferentially affected. Male gender begins to exert a positive effect on growth at a point in gestation where cellular hypertrophy becomes increasingly important. A similar pattern is associated with increasing maternal BMI suggesting, again, an effect on cellular hypertrophy with less of an effect cellular hyperplasia.

The limitations of this analysis should be considered within the context of its methodology. As mentioned above, our study population includes all pregnancies and is not restricted to those that are low-risk. We suggest that this perceived limitation may actually represent an underlying strength to the design, since the data is clinically relevant and for statistical analyses will reduce the likelihood of Type I error. Further, the nature of the reference population, so long as it is broadly representative of general clinical experience, is less of a defining factor with regard to its utility. The reference population is essentially a ‘measuring stick’ for comparisons and ordering of strata within the observation population of interest. Regardless of height being measured in inches or centimeters, a valid comparison and ranking of short versus tall can still be achieved. Our standard population may also be limited by inclusion of only the women who receive a first trimester ultrasound; however we expect that this would be true for only a small number of pregnancies and for the aforementioned reasons this would not impact the use of our standard population for comparison purposes in statistical analysis.

Another potential limitation to our study includes our use of the Hadlock formula for the creation of a metric of fetal size between 16 and 22 weeks, which can be criticized since the formula was not intended for use in that gestational age range. However, we used this as a means of summarizing the individual measurements routinely gathered at that exam into a single metric. While the formula may only imperfectly relate to actual weight at this gestation age range, we suggest that the rank order of fetal size is unlikely to be affected and thus its utility as a reference for comparison is intact.

This analysis has a number of characteristics that may be useful in epidemiological research. The metrics used are, under contemporary obstetrical practice, widely available and constitute standard obstetrical practice. In addition, these metrics can be obtained through a retrospective chart review instead of a more time-consuming prospective data collection. Finally, by allowing a longitudinal examination of fetal size from the first trimester onward, the stage of gestation when differences in growth occur can be determined. More specifically we demonstrate, and parallel contemporary research, that maternal BMI is positively associated with fetal size longitudinally while both fetal gender and maternal smoking are inversely associated with fetal size in a longitudinal manner. Maternal age was not found to influence fetal size in our analysis. In conclusion using available clinical data and in comparison to this reference population, epidemiological studies can examine maternal, fetal, and environmental factors in a way that help to inform mechanistic pathways.

## Supporting Information

S1 Table Means and standard deviations (SD) of ultrasound parameters outside the clinically proscribed window of gestation. (DOCX)
