# Supplementary material for: Utilizing Longitudinal Measures of Fetal Growth to Create a Standard Method to Assess the Impacts of Maternal Disease and Environmental Exposure
Source: PLoS One. 2016 Jan 5;11(1):e0146532. doi: 10.1371/journal.pone.0146532 (PMC4701464; doi:10.1371/journal.pone.0146532)
Supplement: S1 Table — (DOCX) [file pone.0146532.s001.docx]

| S1 Table. Means and standard deviations (SD) of ultrasound parameters outside the clinically proscribed window of gestation. | | | | | | | | | | | | | | |
| --- | --- | --- | --- | --- | --- | --- | --- | --- | --- | --- | --- | --- | --- | --- |
|  | Abdominal diameter (mm) | | Abdominal circumference (mm) | | Biparietal diameter (mm) | | Femur length  (mm) | | Head circumference (mm) | | Occipitofrontal diameter (mm) | | Estimated fetal weight (g) | |
| Gestational age (weeks) | Mean | SD | Mean | SD | Mean | SD | Mean | SD | Mean | SD | Mean | SD | Mean | SD |
| 23 | 60.14 | 3.90 | 188.81 | 12.74 | 56.63 | 2.84 | 42.14 | 2.57 | 211.83 | 9.76 | 73.47 | 3.49 | 643.80 | 85.18 |
| 24 | 63.71 | 4.10 | 200.08 | 13.14 | 59.74 | 3.01 | 44.74 | 2.58 | 223.51 | 10.30 | 77.54 | 3.65 | 748.90 | 98.83 |
| 25 | 67.28 | 4.27 | 211.33 | 13.67 | 62.88 | 3.18 | 47.25 | 2.70 | 235.12 | 10.70 | 81.46 | 3.82 | 867.47 | 115.16 |
| 26 | 70.84 | 4.50 | 222.50 | 14.40 | 65.91 | 3.34 | 49.72 | 2.79 | 246.16 | 11.23 | 85.32 | 4.02 | 998.54 | 135.44 |
| 27 | 74.35 | 4.72 | 233.54 | 15.09 | 68.91 | 3.50 | 52.11 | 2.87 | 256.69 | 11.70 | 88.90 | 4.20 | 1142.42 | 157.47 |
| 28 | 77.81 | 4.92 | 244.46 | 15.47 | 71.67 | 3.63 | 54.37 | 2.96 | 266.39 | 12.09 | 92.30 | 4.40 | 1297.58 | 181.15 |
| 29 | 81.29 | 5.14 | 255.38 | 16.14 | 74.33 | 3.71 | 56.52 | 3.03 | 275.40 | 12.24 | 95.42 | 4.58 | 1464.98 | 206.39 |
| 30 | 84.81 | 5.34 | 266.45 | 16.76 | 76.73 | 3.76 | 58.64 | 3.06 | 283.48 | 12.63 | 98.30 | 4.78 | 1647.07 | 231.23 |
| 31 | 88.23 | 5.60 | 277.20 | 17.60 | 79.12 | 3.85 | 60.68 | 3.14 | 291.04 | 12.96 | 100.95 | 4.93 | 1840.55 | 261.04 |
| 32 | 91.54 | 5.90 | 287.57 | 18.54 | 81.29 | 3.92 | 62.68 | 3.16 | 297.73 | 13.12 | 103.27 | 5.04 | 2041.67 | 295.41 |
| 33 | 94.89 | 6.28 | 298.12 | 19.74 | 83.42 | 4.07 | 64.54 | 3.28 | 304.18 | 13.44 | 105.57 | 5.12 | 2257.84 | 337.89 |
| 34 | 98.13 | 6.51 | 308.28 | 20.46 | 85.33 | 4.18 | 66.38 | 3.28 | 309.88 | 13.45 | 107.63 | 5.19 | 2478.95 | 370.15 |
| 35 | 101.46 | 6.72 | 318.73 | 21.11 | 87.17 | 4.23 | 68.08 | 3.29 | 315.40 | 13.43 | 109.57 | 5.32 | 2711.56 | 402.10 |
| 36 | 104.45 | 6.82 | 328.15 | 21.42 | 88.73 | 4.15 | 69.69 | 3.29 | 319.99 | 12.68 | 111.19 | 5.30 | 2933.74 | 426.69 |
| 37 | 107.30 | 6.94 | 337.09 | 21.80 | 90.02 | 4.12 | 71.19 | 3.29 | 323.66 | 12.39 | 112.53 | 5.30 | 3150.10 | 454.23 |
| 38 | 109.74 | 6.92 | 344.75 | 21.72 | 91.23 | 4.15 | 72.62 | 3.39 | 326.92 | 12.45 | 113.77 | 5.33 | 3349.20 | 460.94 |
| 39 | 112.15 | 6.76 | 352.33 | 21.24 | 92.29 | 4.08 | 73.95 | 3.36 | 329.75 | 12.29 | 114.87 | 5.26 | 3546.91 | 461.59 |
| 40 | 114.25 | 6.72 | 358.93 | 21.12 | 93.11 | 4.05 | 75.17 | 3.36 | 331.88 | 12.07 | 115.71 | 5.20 | 3723.69 | 463.61 |
| 41 | 117.20 | 6.58 | 368.19 | 20.66 | 94.37 | 4.09 | 76.29 | 3.64 | 335.59 | 11.36 | 117.45 | 5.27 | 3968.71 | 474.60 |
| 42 | 118.52 | 6.63 | 372.36 | 20.83 | 94.87 | 4.22 | 76.85 | 3.84 | 337.09 | 11.14 | 118.20 | 5.38 | 4080.21 | 483.72 |
